# The Association of Brachial-Ankle Pulse Wave Velocity with Coronary Artery Disease Evaluated by Coronary Computed Tomography Angiography

**DOI:** 10.1371/journal.pone.0123164

**Published:** 2015-04-13

**Authors:** Hack-Lyoung Kim, Kwang Nam Jin, Jae-Bin Seo, Young Ho Choi, Woo-Young Chung, Sang-Hyun Kim, Myung-A Kim, Joo-Hee Zo

**Affiliations:** 1 Division of Cardiology, Department of Internal Medicine, Boramae Medical Center, Seoul National University College of Medicine, Seoul, Korea; 2 Department of Radiology, Boramae Medical Center, Seoul National University College of Medicine, Seoul, Korea; Universitätsklinikum des Saarlandes, GERMANY

## Abstract

The aim of this study was to investigate whether brachial-ankle pulse wave velocity (baPWV) is associated with the severity of coronary artery disease (CAD) assessed by coronary computed tomography angiography (CCTA), and to evaluate baPWV as a predictor of obstructive CAD on CCTA. A total of 470 patients who underwent both baPWV and CCTA were included. We evaluated stenosis degree and plaque characteristics on CCTA. To estimate the severity of CAD, we calculated the number of segment with plaque (segment involvement score; SIS), stenosis degree-weighted plaque score (segment stenosis score; SSS), and coronary artery calcium score (CACS). The mean baPWV was 1,485 ± 315 cm/s (range, 935-3,175 cm/s). Non-obstructive (stenosis < 50%) and obstructive (stenosis ≥ 50%) CAD was found in 129 patients (27.4%) and 144 (30.6%), respectively. baPWV in patients with obstructive CAD was higher than that of patients with non-obstructive (1,680 ± 396 cm/s versus 1,477 ± 244 cm/s, *P* < 0.001) or no CAD (1,680 ± 396 cm/s versus ± 196 1,389 cm/s, *P* < 0.001). baPWV showed significant correlation with SSS (*r* = 0.429, *P* < 0.001), SIS (*r* = 0.395, *P* < 0.001), CACS (*r* 0.346, *P* < 0.001), and the number of segment with non-calcified plaque (*r* 0.092, *P* = 0.047), mixed plaque (*r* = 0.267, *P* < 0.001), and calcified plaque (*r* = 0.348, *P* < 0.001), respectively. The optimal baPWV cut-off value for the detection of obstructive CAD was 1,547 cm/s. baPWV ≥ 1,547 cm/s was independent predictor for the obstructive CAD. In conclusion, baPWV is well correlated with the severity of CAD evaluated by CCTA. baPWV has the potential to predict severity of coronary artery atherosclerosis.

## Introduction

Arterial stiffness represents arterial aging and arteriosclerosis [1,2]. Increased arterial stiffness is associated with an increased risk of cardiovascular morbidity and mortality [3–7]. Pulse wave velocity (PWV) is a noninvasive method measuring arterial stiffness. PWV is easy to perform, and highly reproducible [8,9]. Therefore, PWV has been widely applied, and played an important role as an indicator of atherosclerotic burden and future cardiovascular events independent of conventional risk factors [3,7,10,11].

With the rapid technical advancements, coronary computed tomography angiography (CCTA) can detect coronary luminal narrowing with excellent image quality. Because of the noninvasive nature and high specificity and negative predictive value of CCTA, it has been commonly used for the evaluation of coronary artery disease (CAD) [12–14]. CCTA also allows the evaluation of plaque characteristics and the calcium amount of the coronary tree, which provide additional information on the coronary risk [15–17].

To date, a few studies using CCTA have demonstrated the association between arterial stiffness and CAD [18–20]. Furthermore, their analyses were relatively simple, mainly focusing on the presence of obstructive CAD or the coronary artery calcium score (CACS) in CCTA. More detailed and comprehensive information on the coronary vasculature obtained by CCTA such as plaque characteristics, segment involvement score (SIS), and segment stenosis score (SSS) has not been assessed in the comparison of arterial stiffness [21,22]. The aim of this study was 1) to investigate whether baPWV is associated with the severity of CAD evaluated by CCTA and 2) to evaluate baPWV as a predictor of obstructive CAD on CCTA.

## Materials and Methods

### Study population

We retrospectively selected a total of 513 patients with suspected CAD who underwent both CCTA and baPWV within 30 days between January 2009 and December 2012. Among these patients, 43 patients were excluded due to following criteria: 1) previous history of myocardial infarction (MI) or revascularization (n = 5), 2) inadequate image quality (n = 21), 3) uncontrolled hypertension (blood pressure >160/100 mmHg) (n = 3), 4) significant peripheral vascular disease (ankle brachial index <0.9) (n = 3), and 5) insufficient clinical information for the study analyses (n = 11). Finally, 470 patients were enrolled in this study. We investigated history of hypertension, diabetes mellitus and dyslipidemia as well as smoking habit. Lipid profiles including total cholesterol, high-density lipoprotein cholesterol, low-density lipoprotein cholesterol and triglyceride levels were also obtained. The pretest probability of CAD was determined by age, sex and the nature of chest pain during initial presentation, and classified as low (<10%), intermediate (10–90%) and high (> 90%) [23].

### Ethics statement

The study protocol was approved by Boramae Medical Center Institutional Review Board (Seoul, Korea, IRB no. # 26-2013-5). The Institutional Review Board waived the need for written informed consent from the participants. All clinical investigation was conducted according to the principles expressed in the Declaration of Helsinki.

### baPWV measurement

Patients were examined in the supine position after ≥ 5 minutes of rest [24]. baPWV were measured using a volume-plethysmographic apparatus (VP-1000; Colin Co. Ltd., Komaki, Japan) in accordance with the manufacturer’s recommendations. Cuffs were wrapped on both upper arm and ankle. Phonogram, pulse volume waveform, blood pressure and heart rate were recorded simultaneously. baPWV was calculated by measuring the time for the pulse wave to travel between the brachial and posterior tibial arteries. The higher value between left and right baPWV was used for study analyses. Patients were taking their regular medications during the measurement.

### CCTA image acquisition

Both coronary artery calcium scoring CT scans and retrospectively ECG-gated coronary CT angiography were performed, using a 64-slice CT scanner (n = 232) (Brilliance 64, Philips Medical Systems, the Netherlands) or 128-slice CT scanner (n = 238) (Ingenuity, Philips Medical Systems, the Netherlands). Patients with a heart rate of 65 beats/min or higher had received bisoprolol 5 to 10 mg orally one hour before the examination if there was no contraindication. Mean heart rate of study patients during the examination was 64.4 ± 9.5 beats/min. Subligual nitroglycerin (0.6 mg) just before the start of CT scan was routinely used. Image acquisition was performed in the cranio-caudal direction. In general, the CT scan covered whole heart from tracheal carina to heart base. A bolus of 70 ml Iomeprol (Iomeron 400, Bracco, Milan, Italy) was administered with an injection rate of 4–5 ml/sec, which was followed by a 40 ml mixture of iodine and saline (ratio 6:4). The scan parameters of 64-slice and 128-slice CT scan were as follows: 64 × 0.625 mm of detector array for both scanner, 420 ms of rotation time for 64-slice CT scanner and 400 ms for 128-slice CT scanner, 120 kV of tube voltage for both scanner, and 800–1,000 mAs of tube current for 64-slice CT scanner and 300–500 mAs for 128-slice scanner. The CT raw data, at 75% of RR interval of the cardiac cycle (mid-diastolic phase), were reconstructed as initial CT data-sets. Additional reconstructions were performed if motion artifacts due to high or irregular heart rate were present. Axial dataset was reconstructed with a slice thickness of 2.5 mm and 1 mm and an increment of 1 mm. We used filtered back projection algorithm for 64-slice CT images and iterative reconstruction algorithm (iDose4, Philips Healthcare, Cleveland, OH) for the 128-slice CT images, respectively. Multi-planar reconstruction images of short-axis, two-chamber, and four-chamber views were routinely reconstructed. The CACS was calculated with the Agatston score using a threshold of 130 Hounsfield units (HU) [25]. We recorded the dose-length product values provided on the CT scanner console. The effective dose of CT scan was calculated as the product of the dose length product multiplied by a conversion coefficient for the chest (k = 0.017 mSv/mGy). Total dose length product of study patients during the examination was 810 ± 358 mGy*cm and effective dose was 13.4 ± 6.4 mSv.

### CCTA image analysis

Two cardiovascular radiologists evaluated CT images with a picture archiving and communication system (PACS) in consensus. They evaluated stenosis degree and plaque characteristics per-segment, according to the modified American Heart Association classification [26]. To estimate the stenosis degree, we visually traced the coronary lumen at the maximal stenotic portion and compared with the mean value of a proximal and distal reference segment. The epicardial coronary artery with luminal stenosis of ≥ 50% was considered as obstructive coronary artery. If a coronary artery segment was uninterpretable even due to heavily calcified plaque or motion artifact, the unevaluable segment was scored similarly to the most proximal segment which was evaluable. Plaques were defined as structures that were clearly distinguished from the lumen and the surrounding pericardial tissue with ≥ 1 mm^2^. Plaques with ≥ 50% of calcified tissue (density ≥ 130 HU) were defined as calcified plaque; plaques with <50% calcium were classified as mixed; and plaques without any calcification were classified as noncalcified plaques [27]. The number of each plaque was assessed in each patient. To evaluate the severity of CAD in each patient, we recorded the presence or number of obstructive coronary artery and overall atherosclerotic plaque burden in the coronary arteries. To assess overall atherosclerotic plaque burden in the coronary arteries, we calculated the SSS and SIS as described in previous studies [21,22]. Based on the degree of stenosis, each coronary artery segment was scored from 0 to 3 (0, none or <30%; 1, 30–49%; 2, 50–69%; 3, ≥ 70%). Then the scores of all 16 individual segments were summed to yield a SSS (range, 0–48). The SIS was calculated as the total number of coronary artery segments having any plaque, irrespective of the stenosis degree (range, 0–16) [21].

### Statistical analysis

Continuous variables were presented as mean ± standard deviation (SD), and categorical variables were expressed as percentages. Spearman’s correlation methods were used for assessing the relationship between baPWV and SSS, SIS, CACS, and number of coronary segment with noncalcified or mixed or calcified plaque. Because the distribution of CACS was positively skewed, and not all of the patients had detectable CAD, the CACS were log-transformed after adding 1 [28]. Mean baPWV values according to the number of major epicardial coronary arteries with significant stenosis (≥ 50%) were compared using analysis of variance (ANOVA). To assess cutoff values of baPWV as a predictor of obstructive CAD on CCTA, receiver-operating characteristic (ROC) curve analysis was used. Multivariable logistic regression analysis was performed to assess independent association between obstructive CAD and baPWV. Age, sex, and history of hypertension, diabetes mellitus and dyslipidemia were adjusted in this model. A *P* value of <0.05 was considered statistically significant. All statistical analyses were conducted using SPSS 18.0 (Chicago, IL, USA).

## Results

### Study population and CCTA findings

The baseline characteristics of the study patients are presented in Table 1. Pretest probability of CAD was low (<10%) in 78 patients (16.6%), intermediate (10%-89%) in 343 (73.0%), and high (≥ 90%) in 49 (10.4%), respectively.

**Table 1:** Baseline characteristics of study patients.

Characteristics	Value (n = 470)
**Age (year)**	58.4 ± 10.4
**Male gender n, (%)**	306 (65.1)
**Body mass index (kg/m** ^**2**^ **)**	24.8 ± 3.3
**Hypertension n, (%)**	221 (47.0)
**Diabetes mellitus n, (%)**	89 (18.9)
**Dyslipidemia n, (%)**	167 (33.5)
**Current smoking n, (%)**	109 (23.2)
**Total cholesterol (mg/dL)**	186 ± 40
**LDL cholesterol (mg/dL)**	116 ± 36
**HDL cholesterol (mg/dL)**	47 ± 11
**Triglyceride (mg/dL)**	120 ± 65
**baPWV (cm/s)**	1,485 ± 315 (range, 935–3,175)

LDL, low density lipoprotein; HDL, high density lipoprotein; baPWV, brachial-ankle pulse wave velocity.

**Fig 1:**
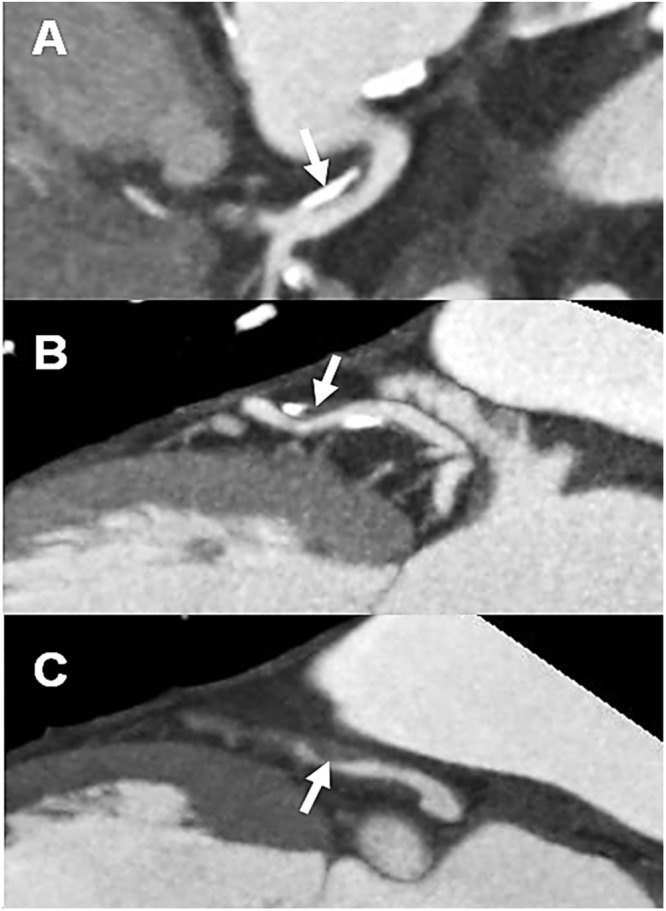
Plaque characteristics of coronary artery on CCTA. Multiplanar reconstruction images demonstrate calcified (A), mixed (B), and noncalcified (C) plaques (arrows) in the proximal left anterior descending coronary arteries. CCTA, coronary computed tomography angiography.

Table 2 demonstrates the CCTA findings. Any atherosclerotic plaque was found in 273 patients (58.1%). About a half of patients (n = 136, 49.8%) had coronary artery plaque in ≤ 5 segments. Obstructive CAD was found in 144 (30.6%). The most common type of plaque composition was calcified plaque (Fig 1A). In persegment analysis, mixed (Fig 1B), noncalcified (Fig 1C), and calcified plaque was found in 128, 130, and 222 segments, respectively. SSS, SIS, CACS was 3.3 ± 4.8 (range, 0–41), 3.6 ± 4.4 (range, 0–13), and 133 ± 354 (range, 0–3,594), respectively.

**Table 2:** Characteristics of CCTA findings.

Characteristics	Number or type	Value
**Coronary artery segment with any plaque**	**≤ 5 segments**	136 (49.8)
	**6–10 segments**	94 (34.4)
	**11–15 segments**	35 (12.8)
	**16–20 segments**	7 (2.6)
	**21–25 segments**	1 (0.4)
	**Total**	273 (58.1)
**Obstructive CAD**	**1 vessel**	55 (38.2)
	**2 vessels**	47 (32.6)
	**3 vessels**	33 (22.9)
	**3 vessels with left main disease**	9 (6.3)
	**Total**	144 (30.6)
**Plaque composition**	**Noncalcified plaque**	0.5 ± 0.9 (0–5)^a^
	**Mixed plaque**	0.5 ± 1.1 (0–8) ^a^
	**Calcified plaque**	1.3 ± 2.0 (0–10)^a^
**Coronary artery calcium score**	**0**	262 (55.7)
	**0.1–100**	95 (20.2)
	**100.1–300**	57 (12.1)
	**> 300**	56 (11.9)

Except where indicated, data are given as number of patients with percentages given in parentheses.

^a^Values expressed as mean number ± standard deviation. Numbers in parentheses are ranges. CCTA, coronary computed tomography angiography; CAD, coronary artery disease.

### The association between baPWV and severity of CAD

**Fig 2:**
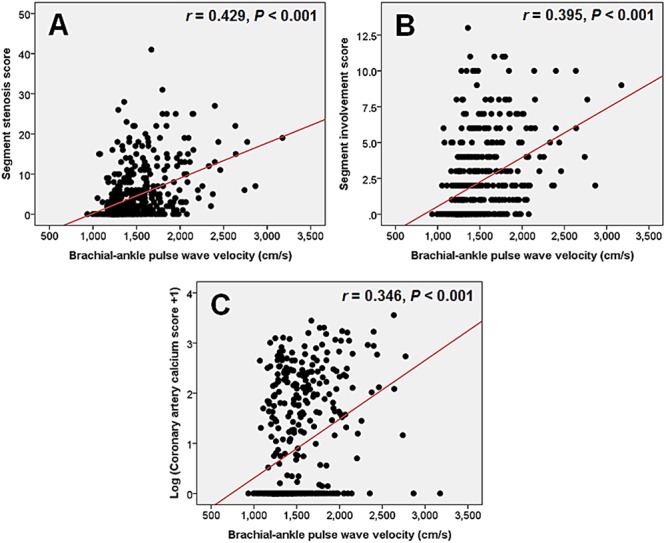
The associations between brachial-ankle pulse wave velocity and severity of coronary artery disease. Graphs show the associations of baPWV with segment stenosis score (A), segment involvement score (B), and coronary artery calcium score (C).

**Fig 3:**
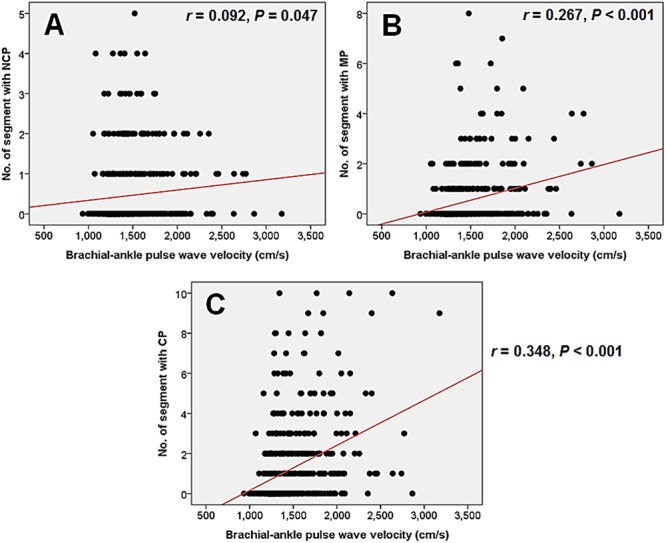
The associations between brachial-ankle pulse wave velocity and plaque characteristics of coronary artery disease. Graphs show the associations of baPWV and the number of coronary segment with noncalcified plaque (A), mixed plaque (B), and calcified plaque (C). NCP, non-calcified plaque; MP, mixed plaque; CP, calcified plaque.

baPWV in patients with obstructive CAD was higher than that of patients with nonobstructive CAD (1,680 ± 396 versus 1,477 ± 244 cm/s, *P*<0.001) and without plaque (1,680 ± 396 versus 1,389 ± 196 cm/s, *P*<0.001). baPWV in patients with nonobstructive CAD was higher than that of patients without plaque (1,477 ± 244 versus 1,389 ± 196 cm/s, *P*<0.001). Mean baPWV was 1,553 ± 315 cm/s in patient with one obstructive CAD (n = 84), 1,682 ± 428 cm/s in those with two (n = 55), 1,717 ± 434 cm/s in those with three (n = 34), and 1,877 ± 195 cm/s in those with three with left main disease (n = 10). In the multiple comparisons with ANOVA, baPWV increased proportionally with increase of the number of obstructed coronary arteries (*P*<0.001). BaPWV had strong positive correlations with SSS (*r* = 0.429, *P*<0.001) (Fig 2A), SIS (*r* = 0.395, *P*<0.001) (Fig 2B), and CACS (*r* = 0.346, *P*<0.001) (Fig 2C). BaPWV also showed significant correlations with the number of coronary segment with noncalcified (Fig 3A), mixed (Fig 3B), and calcified plaque (Fig 3C). Among plaque type, calcified plaque showed highest value of correlation coefficient.

### baPWV as a predictor of obstructive CAD on CCTA

**Fig 4:**
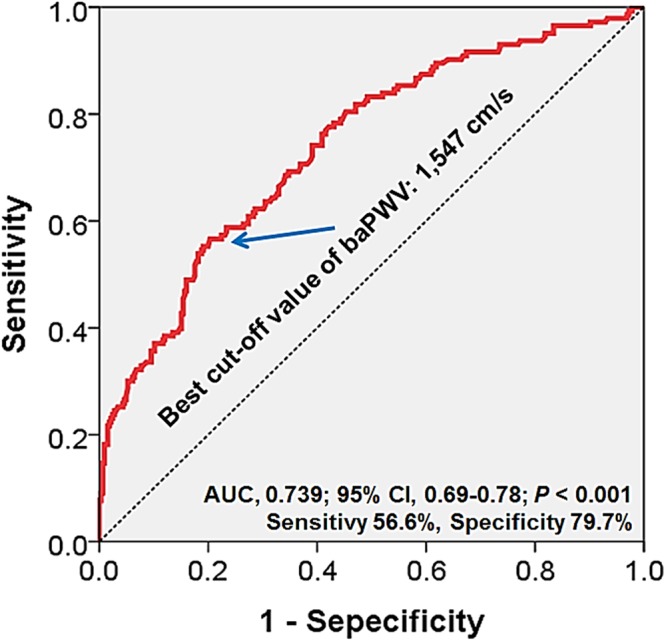
ROC curve analysis identifying best cut-off value of baPWV predicting obstructive CAD. ROC, receiver-operating characteristic; baPWV, brachial-ankle pulse wave velocity; CAD, coronary artery disease; AUC, area under curve; CI, confidence interval.

In the ROC curve analysis, the optimal cut-off value of baPWV for the detection of obstructive CAD was 1,547 cm/s with sensitivity of 56.6% and specificity of 79.7% (area under curve 0.739, 95% confidence interval [CI] 0.69–0.78, *P*<0.001) (Fig 4). In multivariable analysis, baPWV > 1,547 cm/s was an independent predictor of obstructive CAD even after controlling potential confounders including age, sex and history of hypertension, diabetes mellitus and dyslipidemia (odds ratio, 2.56; 95% CI, 1.53–4.28; *P*<0.001) (Table 3).

**Table 3:** Independent predictor of obstructive coronary artery disease on coronary CT angiography.

Variables	OR (95% CI)	*P* values
**Age ≥ 60 years**	2.23 (1.36–3.65)	0.001
**Female sex**	1.51 (0.91–2.48)	0.106
**Hypertension, yes**	1.93 (1.19–3.12)	0.007
**Diabetes, yes**	2.99 (1.72–5.19)	<0.001
**Dyslipidemia, yes**	2.23 (1.40–3.55)	0.001
**baPWV ≥ 1,547 cm/s**	2.56 (1.53–4.28)	<0.001

baPWV, brachial-ankle pulse wave velocity; OR, odds ratio; CI, confidence interval.

## Discussion

The present study showed that baPWV has significant positive correlations with CAD extent, severity and CACS as assessed by CCTA. The predicting power of baPWV for obstructive CAD was independent of age, sex and traditional risk factors, which was confirmed in the multivariable analysis.

Previous studies have shown an association between elevated arterial stiffness and CAD proven by invasive coronary angiography (ICA). High baPWV is an independent risk factor for the presence of significant CAD (≥ 50%) [29–31] as well as coronary artery calcification [30] in patients undergoing ICA. Arterial stiffness was also closely related to CAD severity that was assessed by visual estimation or quantitative methods involving ICA [18,32,33]. Although ICA is still the gold standard for the diagnosis of CAD, its invasiveness and related risks limit its applicability in routine practice [34]. CCTA is a novel noninvasive method that detects CAD with high image quality and excellent diagnostic accuracy as compared to ICA [12–14]. However, CCTA is a relatively new modality, and therefore, there is limited data on the association between arterial stiffness and CAD as proven by CCTA. Recently, a few studies have demonstrated an association between arterial stiffness and CCTA findings. Nam et al. reported that baPWV was associated with CAD and the CACS as detected by CCTA in asymptomatic individuals, and they suggested 1,426 cm/s as an optimal cutoff value of baPWV for obstructive CAD (≥ 50%) [20]. In the other study, Seo at al. demonstrated an association between baPWV and the CACS among high risk patients [19]. Kullo and colleagues documented that aortic PWV is significantly associated with both the presence and quantity of coronary artery calcium, even after controlling for confounders in a community-based analysis [28]. In agreement with these previous findings, our results showed a significant positive correlation between baPWV and CAD in CCTA. As a main strength of this study, when we compared the results obtained in this study to the results of previous studies, a much more comprehensive evaluation using most of the parameters that can be obtained using CCTA, including luminal stenosis severity and extent, CACS and plaque characteristics, was performed to measure CAD.

Several possible mechanisms explaining the association between arterial stiffness and CAD have been suggested [2,3]. The arterial systolic pressure increases and the diastolic pressure decreases in the stiffened artery. Increased systolic pressure causes myocardial wall stress leading to left ventricular hypertrophy, and reduced diastolic pressure causes a decrease of coronary perfusion leading to myocardial ischemia [35]. Also, increased pulse pressure may induce arterial remodeling and the development of plaque and later its rupture [36].

PWV has been known to be associated with cardiovascular risk factors such as old age, hypertension and diabetes mellitus [2]. As expected, there was significant correlation between these risk factors and baPWV in the univariate analyses (data not shown). Therefore, it is important to adjust for these potential compounders that may predict CAD in order to identify the independent relationship between baPWV and the CCTA findings. In the present study, we adjusted for the confounders of age, sex, hypertension, diabetes mellitus and dyslipidemia using the multivariable logistic regression model, and proved that baPWV has an independent association with obstructive CAD in CCTA.

CCTA has been found to be a useful noninvasive tool in the detection of coronary plaque and the investigation of plaque characteristics [15–17]. With CCTA, plaque can be divided according to their characteristics into non-calcified, mixed and calcified plaque with a high degree of accuracy [37,38]. Plaque characteristics proven by CCTA have the power to predict myocardial ischemia and future cardiovascular events [39,40]. The present study firstly showed that baPWV is associated with calcified and mixed plaques but also noncalcificed coronary plaques. However, noncalcified plaque showed lowest value of correlation coefficient among plaque types. It might be in line with previous studies showing an association between arterial stiffness and calcified plaque but not with non-calcified plaque in the carotid artery [41,42]. A possible explanation for our results may be that the same pathophysiological process that causes vessel wall stiffening and calcification. Increased accumulation of fibrous tissue rather than lipid in the arterial wall is associated with not only plaque calcification but also with arterial stiffening [42]. In addition, increased arterial stiffness is considered to result from the long term repetitive cyclic stresses to the arterial wall [1]. It has been documented that arterial stiffness is not affected by early atherosclerosis in an animal model [43]. Therefore, one can expect that arterial stiffness would be more strongly associated with calcified, chronic stabilized, atherosclerotic plaque rather than with unstable fresh plaque. Another explanation is that the presence of calcified plaque in the arterial wall itself my cause stiffness of arterial wall [42]. Further study will be needed to confirm our initial observation.

Noninvasive findings of CAD have played a significant role in screening large populations for the identification of high-risk group. Both PWV and CCTA have been known to have prognostic values [5,27]. Combining the information on PWV and CCTA may strengthen prognostic power for cardiovascular events. Further study will be necessary to reveal that combined information of PWV and CCTA may improve the predictive value for cardiovascular events.

Besides the retrospective design, this study has several limitations. First, this is a cross sectional study, and thus a causal relationship between baPWV and CAD cannot be inferred from our findings. Second, because the study population consisted of patients with suspected CAD, generalization of our results to other people is difficult. Third, we measured arterial stiffness using baPWV rather than carotid-femoral PWV (cfPWV), which is considered as the standard measurement of arterial stiffness. However, the reliability, validity and reproducibility of baPWV have been confirmed in many clinical studies [10,22,33] and in meta-analysis [44]. In addition, good correlation between baPWV and cfPWV is proven [45]. Therefore, baPWV can be considered an acceptable marker of arterial stiffness comparable to cfPWV. Furthermore, compared to cfPWV, measurement of baPWV is easier and simpler. Accordingly, baPWV is more applicable in clinical practice. Lastly, there was no information on medication history that might affect the value of the baPWV or CCTA findings such as statins or renin-angiotensin blockers.

In conclusion, baPWV may be a simple and useful indicator of CAD and could help to identify patients at high risk for CAD.

## Supporting Information

S1 Dataset De-identified minimal dataset. It includes clinical and computed tomographic data of all patients with file format of Microsoft Excel.
(XLSX)
